# Clinical trial evidence of quality-of-life effects of disease-modifying therapies for multiple sclerosis: a systematic analysis

**DOI:** 10.1007/s00415-024-12366-5

**Published:** 2024-04-16

**Authors:** Julian Hirt, Kinga Dembowska, Tim Woelfle, Cathrine Axfors, Cristina Granziera, Jens Kuhle, Ludwig Kappos, Lars G. Hemkens, Perrine Janiaud

**Affiliations:** 1 Research Center for Clinical Neuroimmunology and Neuroscience Basel (RC2NB), University Hospital Basel and University of Basel, Spitalstrasse 2, CH-4031 Basel, Switzerland; 2 Institute of Nursing Science, Department of Health, Eastern Switzerland University of Applied Sciences, St.Gallen, Switzerland; 3 Department of Neurology and MS Center, University Hospital Basel and University of Basel, Basel, Switzerland; 4 Meta-Research Innovation Center at Stanford (METRICS), Stanford University, Stanford, CA USA

**Keywords:** (MeSH): Multiple sclerosis, Quality of life, Patient-reported outcome measures, Randomized controlled trial, Systematic review

## Abstract

**Background:**

Increasingly, patients, clinicians, and regulators call for more evidence on the impact of innovative medicines on quality of life (QoL). We assessed the effects of disease-modifying therapies (DMTs) on QoL in people with multiple sclerosis (PwMS).

**Methods:**

Randomized trials assessing approved DMTs in PwMS with results for at least one outcome referred to as “quality of life” were searched in PubMed and ClinicalTrials.gov.

**Results:**

We identified 38 trials published between 1999 and 2023 with a median of 531 participants (interquartile range (IQR) 202 to 941; total 23,225). The evaluated DMTs were mostly interferon-beta (*n* = 10; 26%), fingolimod (*n* = 7; 18%), natalizumab (*n* = 5; 13%), and glatiramer acetate (*n* = 4; 11%). The 38 trials used 18 different QoL instruments, with up to 11 QoL subscale measures per trial (median 2; IQR 1–3). QoL was never the single primary outcome. We identified quantitative QoL results in 24 trials (63%), and narrative statements in 15 trials (39%). In 16 trials (42%), at least one of the multiple QoL results was statistically significant. The effect sizes of the significant quantitative QoL results were large (median Cohen’s d 1.02; IQR 0.3–1.7; median Hedges’ g 1.01; IQR 0.3–1.69) and ranged between d 0.14 and 2.91.

**Conclusions:**

Certain DMTs have the potential to positively impact QoL of PwMS, and the assessment and reporting of QoL is suboptimal with a multitude of diverse instruments being used. There is an urgent need that design and reporting of clinical trials reflect the critical importance of QoL for PwMS.

**Supplementary Information:**

The online version contains supplementary material available at 10.1007/s00415-024-12366-5.

## Background

Multiple sclerosis (MS) is a chronic, degenerative, and often progressive disease of the central nervous system that can affect multiple parts of the body and result in various symptoms including mobility restrictions, fatigue, pain, depression, and changes in vision and cognition [1]. These symptoms typically lead to a deterioration of the quality of life of persons with MS (PwMS) [2].

While there is no cure yet, key treatment for PwMS includes disease-modifying therapies (DMTs), which target the inflammatory response of the immune system resulting in an almost complete suppression of the disease activity (i.e., suppressing relapses and the occurrence of new lesions in the central nervous system), which in turn would prevent disease progression and may reduce or even avoid the development of disability [3, 4]. With this mechanistic approach in mind and due to the multilayered aspects of the disease activity, numerous clinical trials have evaluated the effects on disability worsening using the Expanded Disability Status Scale (EDSS), relapses, and/or magnetic resonance imaging (e.g., new lesions or brain atrophy) as primary end points [5]. However, patients, clinicians, guideline developers, medical associations, payers, and regulators have increasingly called for more evidence on the impact of innovative medicines for MS on patient-reported outcomes (PROs) [6–8].

PROs are any assessment directly appraised and reported by patients of their health status, such as pain, fatigue, and in particular quality of life (QoL). QoL provides the patient’s unique perspective on both beneficial and harmful treatment effects including treatment burden, symptom alleviation, side effects of treatment and control of disease activity, and increase patients’ involvement in shared decision-making by being key actors in assessing their health. A survey conducted in over 2,000 persons with MS has identified QoL measures, MS symptoms, and preservation of cognition as priority criteria when selecting a DMT [9]. Similarly, a recent initiative developed a patient-centered standard outcome set for MS identifying four domains of interest: disease activity, symptoms, functional status, and QoL [10].

The complexity of the measurement with often multiple domains or subscores and the heterogeneity of instruments being used [5, 11] render comparisons across DMTs difficult and informing treatment decisions hazardous. We aimed to explore which effect estimates on QoL assessed with which instruments are typically obtained in evaluations of DMTs, as a systematic benchmark for an evidence-based research agenda focused on patient-centered end points in MS.

## Methods

We conducted a systematic analysis of clinical trials that assessed the effects of DMTs on QoL in PwMS. To structure our review report, we followed the Preferred Reporting Items for Systematic Reviews and Meta-Analyses (PRISMA) statement [12]. This study was not prospectively registered; and we did not critically appraise the included studies.

### Eligibility criteria

We included randomized controlled trials (RCTs) comparing the effects of approved DMTs with any comparator (e.g., placebo, standard of care, active comparator) on at least one outcome referred to as “quality of life” that reported these effects in a journal article in English language or that were registered in a clinical trial registry with QoL as a prespecified outcome and the trial was categorized as “completed” and/or “has results”. There was no restriction to MS type, disease severity, setting, or year of publication. We considered approved DMTs identified in the European public assessment reports by the European Medicines Agency (EMA) [13] and the Drugs@FDA database by the Food and Drug Administration (FDA) [14] (as of September 29, 2022). We excluded trial protocols and trials labeled as ‘extension’.

### Information sources and search strategy

We searched PubMed (last search: October 4, 2022; Supplementary file 1) using (i) a disease-specific search component (development informed by a recent Cochrane review [15] and several reviews on approved DMTs in MS [16–18]) combined with (ii) identified drug names and corresponding brand names of approved DMTs. Each DMT was transposed into the search strategy by combining a free text word (all fields search and automatic term mapping) with a related Medical Subject Heading (MeSH), if available, e.g., ‘Interferon OR Interferons[MeSH]’. To specifically identify RCTs, we limited the search hits using the PubMed-integrated filter ‘Randomized Controlled Trial’.

We searched ClinicalTrials.gov (last search: April 6, 2023; Supplementary file 1) using the search fields condition (‘Multiple sclerosis’), outcome measure (‘Quality of life’), and intervention (list of identified DMTs as used for the free text PubMed search); limited to trials (‘Interventional’), described as “completed” or “has results”. Registry entries with results were considered for study selection and data extraction. Registry entries of completed trials without posted results were followed to identify journal articles by searching PubMed (all fields search) and Google Scholar using the trial registry number (last search: April 13, 2023).

### Study selection

All full texts of the retrieved journal articles were collected and considered as potentially eligible since the list of outcome measures might be rarely reported in the title and/or abstract. Full texts were screened by one reviewer for eligibility assessment (out of JH, PJ, KD, and TVN) with confirmation of a second reviewer in any unclear case (out of JH, PJ, and LGH).

### Data extraction

One reviewer (out of JH, KD, or PJ) extracted information on trial sample (type of MS, number of randomized participants), intervention and comparator, outcome (number, name, type, hierarchy, and assessor of QoL measures and subscales used, longest follow-up length available, outcome prespecification), design characteristics (blinding and number of trial arms), QoL results (within and between randomized group comparisons; based on registry entries, if available), and bibliographic information (i.e., metadata such as authors, publication year, and journal). Data extraction was based on publication(s) and/or a corresponding registry entry using an electronic spreadsheet.

### Data analysis

We summarized the trial characteristics using descriptive statistics. We considered all individual subscales of QoL instruments as QoL measures. QoL instruments were categorized as generic or disease-specific measures according to the description of the QoL instrument. Symptom-specific QoL instruments were categorized as disease-specific measures.

We considered comparative effects (i.e., between randomized groups) as QoL results. Data on QoL not reflecting effects of treatments (e.g., before–after changes within study arms) were not considered. We stratified QoL results by type of outcome measure (i.e., disease-specific or general QoL measure). We categorized QoL results into (1) quantitative QoL effects (between-group differences with dispersion were reported, e.g., mean differences with standard deviation or data on change from baseline to follow-up were reported that allowed us to calculate between-group differences); (2) QoL effects with *p* value alone (not providing data to derive quantitative effects); (3) narrative statements on between-group differences. To assess the statistical significance of the QoL results, we used reported *p* values (with *p* < 0.05 indicating statistical significance), reported or self-calculated confidence intervals (CI; with confidence interval of a mean difference not crossing the null indicating statistical significance), or statements by authors declaring the results as “statistically significant”. To allow the comparison of effects sizes across trials, whenever possible, we converted quantitative QoL effects to Cohen’s d and Hedges’ g. We also report the proportion of effect size superior to the 0.2 (Hedges’ g) minimal clinically important differences (MCID) as defined by current guidelines for health-technology assessment and reimbursement decisions on quality of life assessments [19]. We used R (version 4.2.2) for data analysis.

## Results

We identified 38 eligible trials that reported QoL results in 40 publications between 1999 and 2023 (median publication year: 2015; Table 1; Supplementary file 2), with 34 trials (89%) published in journal articles [20–50] and 4 trials (11%) registered on ClinicalTrials.gov [51–54].

**Table 1:** Summary characteristics of trials reporting on QoL effects of DMTs (n = 38)

	*n* (%)
Publication year	
1999–2009	10 (27)
2010–2019	23 (61)
2020–2023	5 (13)
Number of randomized participants (M; IQR; range; total)	531; 203–612; 13–1,516; 23,255
Blinding ***	
Double	23 (61)
Open	14 (37)
Single	1 (3)
Type of MS	
Relapsing–remitting	24 (63)
Multiple	7 (18)
Secondary progressive	4 (11)
Primary progressive	1 (3)
Clinically isolated syndrome	1 (3)
Pediatric onset	1 (3)
Experimental DMT *	
Interferon	10 (26)
Fingolimod	7 (18)
Natalizumab	5 (13)
Glatiramer acetate	4 (11)
Dimethyl fumarate	3 (8)
Ocrelizumab	3 (8)
Alemtuzumab	2 (5)
Ozanimod	2 (5)
Other **	3 (6)
Number of trial arms (M, IQR, range)	2, 2–3, 2–4
QoL outcomes prespecified (trial registry)	18 (47)
QoL results posted (trial registry)	15 (39)
Follow-up length ****	
3 m	3 (8)
6 m	7 (18)
12 m	4 (11)
18 m	2 (5)
24 m	20 (53)
36 m	2 (5)

*More than one category possible

**Single counts; see Supplementary file 2 for details

***For blinding of outcome assessment, see Table 3

**** ± 4 weeks

*DMT *disease-modifying therapy, *IQR *interquartile range, *m *months, *M *median, *MS *multiple sclerosis, *n *number, *QoL *quality of life

### Trial characteristics

The 38 trials had a median of 531 participants (interquartile range (IQR) 202 to 941; total 23,225 PwMS). Studies were double blinded (*n* = 23, 61%), single blinded (*n* = 1, 3%), or open labeled (*n* = 14, 37%). Twenty-four trials (63%) included PwMS with relapsing–remitting MS only, 7 trials (18%) included people with multiple types of MS, and 4 trials (11%) included only participants with secondary progressive MS (*n* = 1;3%). Three trials included either only participants with primary progressive MS (*n* = 1; 3%), clinically isolated syndrome (*n* = 1; 3%), or pediatric-onset MS (*n* = 1; 3%). The trials evaluated 13 different DMTs, mostly interferon-beta (*n* = 10; 26%), fingolimod (*n* = 7; 18%), natalizumab (*n* = 5; 13%), and glatiramer acetate (*n* = 4; 11%); and trials evaluated two to four arms (median: 2; IQR: 2 to 3). Only 18 trials (48%) prespecified the QoL outcomes in a clinical trial registry, with 15 trials (40%) that posted results in the registry. Follow-up was 3–36 months (22 trials had a follow-up ≥ 24 months; 58%; Tables 1, 2).

**Table 2:** Characteristics of 38 trials reporting on QoL effects of DMTs

Trial acronym, year	Type of MS	N	Intervention and comparison	QoL measures (n) *	Trial arms (n)	QoL results (n)	Follow-up **
NOVA [47], 2023	Relapsing–remitting	499	Natalizumab 6-week dosing vs. natalizumab 4-week dosing	4	2	4	18m
CONNECT [48], 2022	Pediatric onset	156	Interferon beta-1a vs dimethyl fumarate	8	2	8	24m
SUNBEAM [26], 2021	Multiple	1.346	Lower vs higher dose of ozanimod vs interferon beta-1a	2	3	6	12m
MOVING [20], 2020	Multiple	13	Interferon beta-1b vs fingolimod	1	2	1	6m
RELIEF [43], 2020	Not specified	200	Morning vs evening administration of interferon-beta 1a	1	2	1	3m
CONFIDENCE [25], 2019	Relapsing–remitting	861	Higher vs lower frequency of glatiramer acetate administration	2	2	2	6m
GATEWAY II [31], 2019	Multiple	55	Rituximab–glatiramer acetate vs placebo and rituximab–glatiramer acetate	2	2	2	24m
RADIANCE [23], 2019	Multiple	1.320	Interferon beta-1a vs ozanimod	3	3	6	24m
ASCEND [33], 2018	Secondary progressive	889	Natalizumab vs placebo	1	2	1	24m
TOWER [45], 2018	Relapsing–remitting	1.165	Lower vs higher dose of teriflunomide vs placebo	2	3	6	12m
CARE-MS I [21], 2017	Relapsing–remitting	563	Interferon beta-1a vs alemtuzumab	5	2	5	24m
CARE-MS II [21], 2017	Relapsing–remitting	628	Interferon beta-1a vs alemtuzumab	5	2	5	24m
GOLDEN [53], 2017	Relapsing–remitting	151	Interferon beta 1b vs fingolimod	2	2	2	18m
OPERA I [30], 2017	Relapsing–remitting	821	Ocrelizumab vs interferon beta-1a	1	2	1	24m
OPERA II [30], 2017	Relapsing–remitting	835	Ocrelizumab vs interferon beta-1a	1	2	1	24m
ORATORIO [39], 2017	Primary progressive	732	Ocrelizumab vs. placebo	1	2	1	24m
ADVANCE [40], 2015	Relapsing–remitting	1.516	Peginterferon vs placebo	6	3	18	24m
EPOCa [51], 2015	Relapsing–remitting	61	DMT vs Fingolimod	2	2	2	6m
GLACIER [49], 2015	Relapsing–remitting	209	Lower vs higher dose of glatiramer acetate	1	2	2	3m
NA [37], 2015	Not specified	69	Interferon beta-1a (Avonex and Rebif) vs interferon beta-1b (Betaferon)	2	3	6	12m
CONFIRM [35], 2014	Relapsing–remitting	1.417	Dimethyl fumarate two times daily vs dimethyl fumarate three times daily vs glatiramer acetate vs placebo	5	4	30	24m
DEFINE [32], 2014	Relapsing–remitting	1.237	Dimethyl fumarate two times daily vs dimethyl fumarate three times daily vs placebo	5	3	15	24m
EPOCb [52], 2014	Relapsing–remitting	298	DMT vs fingolimod	2	2	2	6m
EPOCc [27], 2014	Not specified	1.053	Injectable DMT vs fingolimod	2	2	2	6m
FREEDOMS II [22], 2014	Relapsing–remitting	1.083	Fingolimod vs placebo	3	3	9	24m
NA [50], 2014	Relapsing–remitting	19	De-escalating natalizumab to interferon beta 1b vs continued natalizumab	2	2	2	12m
MSCRG [36], 2011	Not specified	301	Interferon beta-1a vs placebo	3	2	3	24m
NA [28], 2001	Not specified	718	Interferon beta-1b vs placebo	1	2	1	36m
NA [38], 2011	Multiple	281	Lower vs higher dose of fingolimod vs placebo	1	3	3	6m
RebiQoL [54], 2009	Not specified	232	Rebif new formulation non-titrated vs Rebif new formulation titrated	3	2	3	3m
AFFIRM [46], 2007	Not specified	942	Natalizumab vs placebo	3	2	3	24m
NA [44], 2007	Multiple	231	Intravenous immunoglobulin vs placebo	1	2	1	24m
SENTINEL [46], 2007	Not specified	1.171	Natalizumab vs placebo in addition to Interferon beta-1a	3	2	3	24m
BENEFIT [34], 2006	Clinically isolated syndrome	487	Interferon beta-1b vs placebo	2	2	2	24m
NA [42], 2004	Not specified	939	Lower vs higher dose of interferon-beta 1b vs. placebo	1	3	3	36m
IMPACT [24], 2002	Not specified	436	Interferon beta-1a vs placebo	11	2	11	24m
MIMS [29], 2002	Multiple	194	Lower vs higher dose of mitoxantrone vs placebo	1	3	3	24m
NA [41], 1999	Relapsing–remitting	97	Interferon alfa-2a vs placebo	9	3	27	6m

*Including subscales

** ± 4 weeks

*m *months, *MS* multiple sclerosis, *n *number, *NA *not available, *QoL *quality of life, *vs *versus

### QoL measure characteristics

The 38 trials used 110 QoL measures or subscales of measures (a median of 2 QoL-measures per trial; IQR 1–3; range 1–11), collected by 18 different QoL instruments (9 generic QoL instruments, 8 disease-specific QoL instruments, and 1 QoL instrument that covers both generic and disease-specific subscales). A generic QoL measure was used in 29 trials (76%), mostly the Short Form 36 (SF-36; *n* = 18; 47%) and the European Quality of Life 5 Dimension (EQ-5D; *n* = 9; 24%). A disease-specific QoL measure was used in 19 trials (50%), mostly the Functional Assessment of Multiple Sclerosis (FAMS; *n* = 4; 11%), the Multiple Sclerosis Impact Scale-29 (MSIS-29; *n* = 4; 11%), and the Multiple Sclerosis Quality of Life-54 (MSQOL-54; *n* = 4; 11%). QoL was never the single primary outcome, only two trials (5%) used it as a co-primary outcome, and most often was a secondary outcome (*n* = 23; 61%). In most trials, QoL outcomes were assessed by patients themselves (*n* = 25; 69%); in 1 trial (3%) by parents of affected children and no detailed information on outcome assessment was reported in 15 trials (40%). Outcome assessment was mostly blinded (*n* = 23 trials; 61%; Table 3).

**Table 3:** Characteristics of 110 QoL measures used in all 38 trials

	Trials *n* (%)	QoL measures *n* (%)
Total	38 (100)*	110 (100)
Type of measure**		
Generic instruments	29 (76)	78 (71)
SF-36	18 (47)	38 (35)
EQ-5D	9 (24)	15 (14)
PedsQL	1 (3)	8 (7)
MSQLI	2 (5)	4 (4)
GHQ VAS	4 (11)	4 (4)
SIP	2 (5)	4 (4)
SF-12	1 (3)	2 (2)
Other***	3 (8)	3 (3)
Disease-specific instruments	19 (50)	32 (29)
MSIS-29	4 (11)	7 (6)
FAMS	4 (11)	4 (4)
MSQOL-54	4 (11)	8 (7)
MSQLI	3 (8)	8 (7)
Other ***	5 (13)	5 (5)
Trial outcome hierarchy of QoL		
Primary	0 (0)	0 (0)
Co-primary	2 (5)	3 (3)
Secondary	23 (61)	53 (48)
Tertiary	5 (13)	21 (19)
Exploratory/other	3 (8)	5 (5)
Not specified	7 (18)	28 (26)
Assessor		
Patient	25 (69)	65 (59)
Proxy	1 (3)	4 (4)
No information	15 (39)	41 (37)
Blinded outcome assessment		
Yes	23 (61)	36 (33)
No	14 (37)	36 (33)
No information	1 (3)	38 (35)

*More than one category possible

**A QoL measure may appear in both generic and disease-specific type of measure, since subscales are not considered in this table; see Supplementary file 2 for details

***Single counts; see Supplementary file 2 for details

*EQ-5D *European Quality of Life 5 Dimensions, *FAMS *Functional Assessment of Multiple Sclerosis, *GHQ *General Health Questionnaire, *MSIS *Multiple Sclerosis Impact Scale, *MSQLI *Multiple Sclerosis Quality of Life Inventory, *MSQOL *Multiple Sclerosis Quality of Life, *n *number, *PedsQL *Pediatric Quality of Life Inventory, *SF *Short Form, *SIP *Sickness Impact Profile, *VAS *visual analog scale

### Reporting of quality-of-life results

Because multiple trials had more than two arms and used multiple QoL measures, there were up to 30 QoL results per trial with a total of 203 QoL results across the 38 trials (median of 3 per trial; IQR 2–6; range 1–30).

We identified quantitative QoL results in 24 out of the 38 trials (63%; 89 of 203 QoL results), QoL effects with p value alone in 8 trials (21%; 27 of 203 QoL results), and narrative statements in 15 trials (39%; 57 out of 203 QoL results). All trials reported at least one QoL result (85%; 173 of 203 QoL results), but not all results were reported, 30 of 203 QoL results (15%) were prespecified in the registry and/or mentioned in the article, but we identified no data (6 of 38 trials; 16%; Table 4).

**Table 4:** Reporting of QoL results and impact of DMT on QoL (*n* = 203 QoL results in *n* = 38 included trials)

	QoL results per trial *n* (%)	QoL results across trials *n* (%)		
	Total 38 (100)*	Total 203 (100)	Generic QoL measure 149 (73)	Disease-specific QoL measure 54 (27)
REPORTING of QoL results				
Reported results	32 (84)	173 (85)	120 (81)	53 (98)
Quantitative results	24 (63)	89 (44)	46 (31)	43 (80)
* p* value alone	8 (21)	27 (13)	25 (17)	2 (4)
Narrative statements (without *p* value)	15 (39)	57 (29)	49 (33)	8 (15)
Not all results reported	6 (16)	30 (15)	29 (20)	1 (2)
IMPACT of DMT on QoL				
Statistically significantly favors experimental DMT **	16 (42)	49 (24)	31 (21)	18 (33)
Quantitative results	9 (24)	27 (13)	11 (5)	16 (30)
* p* value alone	5 (13)	17 (8)	15 (8)	2 (4)
Narrative statements (without *p* value)	4 (11)	5 (2)	5 (2)	0 (0)
Statistically significantly favors control	1 (3)	1 (1)	0 (0)	1 (2)
Quantitative results	1 (3)	1 (1)	0 (0)	1 (2)
No group difference	29 (76)	123 (61)	89 (60)	34 (63)
Quantitative results	18 (47)	61 (30)	35 (18)	26 (48)
p value alone	6 (16)	10 (5)	10 (5)	0 (0)
Narrative statements (without p value)	12 (32)	52 (26)	44 (22)	8 (15)

*More than one category per trial possible

**Experimental and control has not been defined in one trial [37]

*DMT *disease-modifying therapy, *n *number, *QoL *quality of life

### Impact of DMTs on QoL

In 16 trials (42%), for at least one of the multiple QoL results, a statistically significant result was indicated which always favored the experimental DMT (*n* = 16 trials, 42%) with the exception of one QoL result in 1 trial (3%) where placebo was favored (d − 0.5; 95% CI − 0.66 to − 0.34, [42]; Supplementary file 1). Only six trials (16%) had statistically significant results in favor of the experimental DMT across all QoL results (comparisons and subscales) reported for the respective trials.

**Fig. 1:**
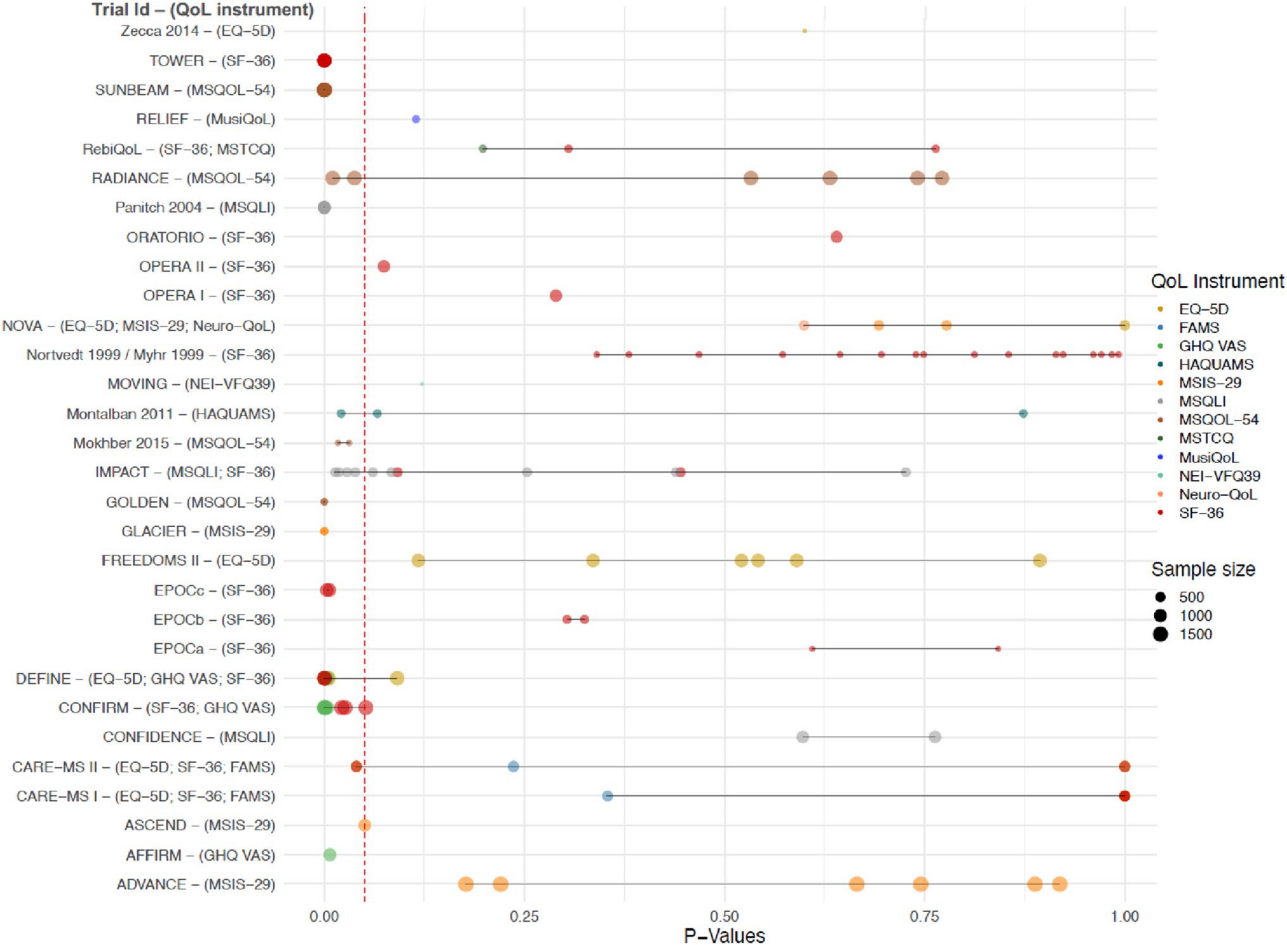
Distribution of *p* values (116 QoL results from 30 trials). Note: The 116 QoL results come from 89 quantitative results and 27 reported as *p* values alone. Abbreviations: *DMT *disease-modifying therapy, *EQ-5D *European Quality of Life Dimensions, *FAMS *Functional Assessment of Multiple Sclerosis, *GHQ VAS *General Health Questionnaire Visual Analog Scale, *HAQUAMS *Hamburg Quality of Life Questionnaire for Multiple Sclerosis, *MSIS *Multiple Sclerosis Impact Scale, *MSQLI *Multiple Sclerosis Quality of Life Inventory, *MSQOL *Multiple Sclerosis Quality of Life, *MSTCQ *Multiple Sclerosis Treatment Concerns Questionnaire, *MusiQoL *Multiple Sclerosis International Quality of Life, *n *number, *NEI-VFQ *National Eye Institute Visual Functioning Questionnaire, *QoL *quality of life, *SF *Short Form

Out of all 173 reported QoL results, 50 were statistically significant and 123 were not (cases with quantitative results: 28 statistically significant and 61 with no group differences; cases with p values alone: 17 statistically significant and 10 with no group differences; and cases with narrative statements: 5 statistically significant and 52 with no group differences; Table 4; Fig. 1). Among the 89 with quantitative results, 28 point estimates of the Hedges' g were larger than the 0.2 MCID threshold, of which 24 were statistically significant. Conversely, four of the statistically significant Hedges' g did not reach the MCID threshold.

In the nine trials (24%) with significant quantitative QoL results, the effect sizes of DMTs on QoL were large (median Cohen’s d 1.02; IQR 0.3–1.7; median Hedges’ g 1.01; IQR 0.3–1.69) and ranged between d 0.14 and 2.91 (Supplementary file 1).

## Discussion

Our systematic search found 38 trials that reported the effects of DMTs on QoL outcomes in PwMS, however, rarely being the primary outcome. The trials used many different QoL subscales that were collected by multiple generic and MS-specific QoL instruments. Almost half of all trials reported at least one statistically significant QoL effect out of the multiple subscales and comparisons reported by the respective trials. This agrees with a review from 2017 which found for earlier studies that positive effects of DMTs are reported across very different scales of health related QoL [17]. However, the number of trials with statistically significant results across all QoL results reported for the respective trials was limited, only 6 out of the 38 trials. Furthermore, out of the 203 QoL results reported across all trials, only 50 were statistically significant—statistically significant results on some subscales coexisted with non-significant results on others, sometimes even within the same instrument.

While we did not systematically assess the quality of reporting, e.g., by using the CONSORT extension for PROs [55], we observed that results on QoL were not consistently reported across all trials and within trials. The inability to derive quantitative results from the reported data for almost half of our comparisons is indicative of substantial reporting deficiencies within this field and may indicate strong reporting bias. This observation is supported by a recent meta-epidemiological analysis showing the inadequate reporting of QoL in neuroscience [56]. In addition, with the inconsistencies in reporting effects within trials, one cannot exclude the possibility of selective reporting of results. For example, within a trial, not all QoL results were reported or were reported with different level of details (e.g., quantitatively for some and narratively for others). While our work cannot replace a thorough assessment of the full body of evidence on the multiple clinical questions the 38 studies aimed to address, some issues are noteworthy. As outlined above, we observed substantial reporting deficits with risk of reporting bias, the lack of blinding in some cases, and the presence of small sample sizes with imprecise effect estimates, which would all likely reduce the certainty of evidence regarding the effect of DMTs on QoL in Grades of Recommendation, Assessment, Development and Evaluation (GRADE) assessments and subsequent recommendations in clinical guidelines [57]. This highlights the urgent need for improvement of the research agenda and implications of these findings to improve not only on research, but also clinical decisions.

QoL is a subjective measure highly dependent on individual patient factors (e.g., mood) and external factors (e.g., socioeconomic status) [58]. Instruments and even subscales might have different meaning for different patients. This could explain the profusion of existing instruments and subscales as illustrated by our results and also highlighted in a 2017 systematic review, which identified 402 PROs used in MS observational studies and randomized trials (RCTs), of which 82 were MS specific and 10 focused specifically on QoL [11]. The choice of the instrument and its fit for purpose is therefore even more essential, yet many instruments used in MS have poor content validity [11, 59]. The complexity in the choice of the instrument and its interpretation most likely also explain why they only play a peripheral role in trials assessing DMTs in PwMS [5, 60] being mostly assessed as secondary or exploratory outcomes, as shown by our results. Overall, the multitude in subscales with the heterogeneity of results and inconsistent reporting makes it currently very often difficult to infer a clinical decision regarding the impact of DMTs on health-related quality of life.

Late 2022, a patient-centered outcome set according to the COMET initiative was developed recommending using MSIS-29. Our analysis provides a baseline benchmark that may allow to assess its uptake [10].

### Limitations

Our work has some limitations. First, we did not assess the quality of the evidence and the risk of bias, but we assessed the study design characteristics, of which some indicate quality (sample size to provide precise effects) and risk of bias (e.g., blinding in a scenario of assessing subjective patient-reported outcomes).

Second, we also have not conducted an exhaustive search in multiple databases with very sensitive search filters; however, we have conducted a complete assessment of all full texts (not only relying on abstract information as in a typical systematic review) and complemented the search with extensive assessment of clinical trial registries. We considered QoL results that were reported in peer-reviewed journal articles. We did not consider trial protocols. There might be more results on QoL outcomes that have been prespecified in trial protcols, but not reported in the publication of trial results. If QoL outcomes were not mentioned in the publication of trial results or in the trial registry, we would have overlooked them. We clearly assume a lack of reporting of QoL results. This would mean that the underreporting and selective reporting of results were underestimated in our sample.

Third, eligible trials were selected and extracted by only one reviewer. However, as we directly searched all full texts whether they assessed and reported the impact of DMTs on QoL and the involved reviewers were experienced in these methods, we assume that this did not lead to considerable data errors.

Fourth, QoL measurements are often measured longitudinally with multiple time points, yet we only extracted and assessed the measurement with the longest follow-up available. QoL measurements may also be prone to response shift, whereby the individual’s reference of well-being changes overtime [61], potentially impacting estimation of QoL in clinical trials with longitudinal follow-up [62].

Finally, this is not a systematic review on the benefits and harms of all the DMTs to inform treatment decisions, but a meta-research survey on one type of outcome to inform further research on QoL in MS. Beyond the empirical information provided by this study, further important characteristics of the instruments need to be considered in the selection of quality-of-life instruments, such as the psychometric properties, ease of administration, and domains covered. QoL assessment and reporting is suboptimal, but is optimal to inform patient-relevant decision-making. Uncertainties remain, but gaps can only be filled by having QoL systematically assessed and reported. More insight into the treatment effect sizes can help by fostering sample size calculation by providing the range of effect sizes that can be expected. With the rise of digital health measures, assessment of QoL might gain in granularity and efficiency, opening new horizons toward a more personalized QoL assessment.

## Conclusions

Our results indicate the potential of certain DMTs to positively impact QoL of PwMS. However, there seems to be no generally accepted standard for assessing disease-specific QoL in PwMS, and rarely is this end point in the focus of DMT-evaluating trials. The critical importance of QoL for PwMS urgently needs to be better reflected in the design, registration, and reporting of future MS clinical trials. This meta-research survey serves as a valuable resource for researchers, clinicians, and policymakers, promoting a deeper understanding of the interplay between DMTs and QoL in the context of MS.

### Supplementary Information

Below is the link to the electronic supplementary material.

Supplementary file1 (PDF 1092 KB)

Supplementary file2 (XLSX 67 KB)

## Data Availability

Data generated and analyzed in this study is provided in Supplementary file 1 and 2.
