# Supplementary material for: Clinical trial evidence of quality-of-life effects of disease-modifying therapies for multiple sclerosis: a systematic analysis
Source: J Neurol. 2024 Apr 16;271(6):3131–41. doi: 10.1007/s00415-024-12366-5 (PMC11136790; doi:10.1007/s00415-024-12366-5)
Supplement: Supplementary file 1 — Supplementary file1 (PDF 1092 KB) [file 415_2024_12366_MOESM1_ESM.pdf]

APPENDIX

Appendix 1. Search strategy

PubMed: October 4, 2022; 941 hits

Search	Query
#1	("Multiple Sclerosis"[Mesh:noexp] OR "Multiple Sclerosis, Chronic Progressive"[Mesh] OR "Multiple Sclerosis, Relapsing-Remitting"[Mesh] OR "Demyelinating Diseases"[Mesh:noexp] OR "Optic Neuritis"[Mesh] OR "Demyelinating Autoimmune Diseases, CNS"[Mesh:noexp] OR "Encephalomyelitis, Acute Disseminated"[Mesh] OR "Myelitis, Transverse"[Mesh] OR "multiple sclerosis"[Tiab] OR "neuromyelitis optica"[Tiab] OR "optic neuritis"[Tiab] OR "devic disease"[Tiab] OR "demyelinating disease"[Tiab] OR adem[Tiab] OR "demyelinating disorder"[Tiab] OR "clinically isolated syndrome"[Tiab] OR "transverse myelitis"[Tiab] OR "acute disseminated encephalomyelitis"[Tiab] OR "encephalomyelitis"[Tiab])
#2	(Interferon OR Interferons[Mesh] OR peginterferon OR glatiramer "Glatiramer Acetate"[Mesh] OR alemtuzumab OR Alemtuzumab[Mesh] OR daclizumab OR Daclizumab[Mesh] OR mitoxantrone OR Mitoxantrone[Mesh] OR fingolimod OR "Fingolimod Hydrochloride"[Mesh] OR "Dimethyl fumarate" OR "Dimethyl Fumarate"[Mesh] OR Teriflunomide OR ocrelizumab OR natalizumab OR natalizumab[Mesh] OR rituximab OR Rituximab[Mesh] OR Aubagio OR Copaxone OR Avonex OR "Interferon beta-1a"[Mesh] OR Mavenclad OR Lemtrada OR Alemtuzumab[Mesh] OR Rebif OR Betaferon OR "Interferon beta-1b"[Mesh] OR Plegridy OR Extavia OR Zeposia OR Redimune OR Privigen OR "Immunoglobulins, Intravenous"[Mesh] OR Gilenya OR Gilenia OR "Fingolimod Hydrochloride"[Mesh] OR Tascenso OR Tecfidera OR Ocrevus OR Tysabri OR Mabthera OR Interferon-beta OR "Interferon-beta"[Mesh] OR IFN-beta-1b OR IFN-beta-1a OR Betaseron OR Peg-IFNbeta-1a OR Novatrone OR Zinbryta OR Daclizumab[Mesh] OR Cladribine OR Cladribine[Mesh] OR Leustatin OR Mayzent OR siponimod OR Ponvory OR ponesimod OR fampridine OR Fampyra OR Vumerity OR "diroximel fumarate" OR Kesimpta OR ofatumumab OR ozanimod OR Nerventra OR laquinimod OR Actimmune OR "Alferon N Injection" OR "interferon gamma-1b" OR "Interferon-gamma"[Mesh] OR Besremi OR "ROPEGINTERFERON ALFA-2B-NJFT" OR Intron A OR "INTERFERON ALFA-2B" OR "Interferon alpha-2"[Mesh] OR Pegasys OR "PEGINTERFERON ALFA-2A" OR Pegintron OR "PEGINTERFERON ALFA-2B" OR Sylatron OR Glatopa OR Campath OR TERIFLUNOMIDE OR Riabni OR "RITUXIMAB-ARRX" OR Rituxan OR Ruxience OR "RITUXIMAB-PVVR" OR Truxima OR "RITUXIMAB-ABBS" OR Ampyra OR Dalfampridine OR "4-Aminopyridine"[Mesh] OR Firdapse OR "AMIFAMPRIDINE PHOSPHATE" OR Ruzurgi OR "AMIFAMPRIDINE" OR amifampridine[Mesh] OR Arzerra)
#3	#1 AND #2
#4	#3 Filters: Randomized Controlled Trial

ClinicalTrials.gov: April 6, 2023 (Beta version: https://beta.clinicaltrials.gov/); 57 hits

Condition:
Multiple sclerosis

Outcome measure:
Quality of life

Intervention:
Interferon OR Interferons OR peginterferon OR glatiramer "Glatiramer Acetate" OR alemtuzumab OR Alemtuzumab OR daclizumab OR Daclizumab OR mitoxantrone OR Mitoxantrone OR fingolimod OR "Fingolimod Hydrochloride" OR "Dimethyl fumarate" OR "Dimethyl Fumarate" OR Teriflunomide OR ocrelizumab OR natalizumab OR natalizumab OR rituximab OR Rituximab OR Aubagio OR Copaxone OR Avonex OR "Interferon beta-1a" OR Mavenclad OR Lemtrada OR Alemtuzumab OR Rebif OR Betaferon OR "Interferon beta-1b" OR Plegridy OR Extavia OR Zeposia OR Redimune OR Privigen OR "Immunoglobulins, Intravenous" OR Gilenya OR Gilenia OR "Fingolimod Hydrochloride" OR Tascenso OR Tecfidera OR Ocrevus OR Tysabri OR Mabthera OR Interferon-beta OR "Interferon-beta" OR IFN-beta-1b OR IFN-beta-1a OR Betaseron OR Peg-IFNbeta-1a OR Novatrone OR Zinbryta OR Daclizumab OR Cladribine OR Cladribine OR Leustatin OR Mayzent OR siponimod OR Ponvory OR ponesimod OR fampridine OR Fampyra OR Vumerity OR "diroximel fumarate" OR Kesimpta OR ofatumumab OR ozanimod OR Nerventra OR laquinimod OR Actimmune OR "Alferon N Injection" OR "interferon gamma-1b" OR "Interferon-gamma" OR Besremi OR "ROPEGINTERFERON ALFA-2B-NJFT" OR Intron A OR "INTERFERON ALFA-2B" OR "Interferon alpha-2" OR Pegasys OR "PEGINTERFERON ALFA-2A" OR Pegintron OR "PEGINTERFERON ALFA-2B" OR Sylatron OR Glatopa OR Campath OR TERIFLUNOMIDE OR Riabni OR "RITUXIMAB-ARRX" OR Rituxan OR Ruxience OR "RITUXIMAB-PVVR" OR Truxima OR "RITUXIMAB-ABBS" OR Ampyra OR Dalfampridine OR "4-Aminopyridine" OR Firdapse OR "AMIFAMPRIDINE PHOSPHATE" OR Ruzurgi OR "AMIFAMPRIDINE" OR amifampridine OR Arzerra

146 hits

Filter for “interventional”: 98 hits
- Filter for “with results”: **36** hits
- Filter for “without results” (62 hits) and “completed”: **21** hits

Appendix 2. Literature search and study selection process

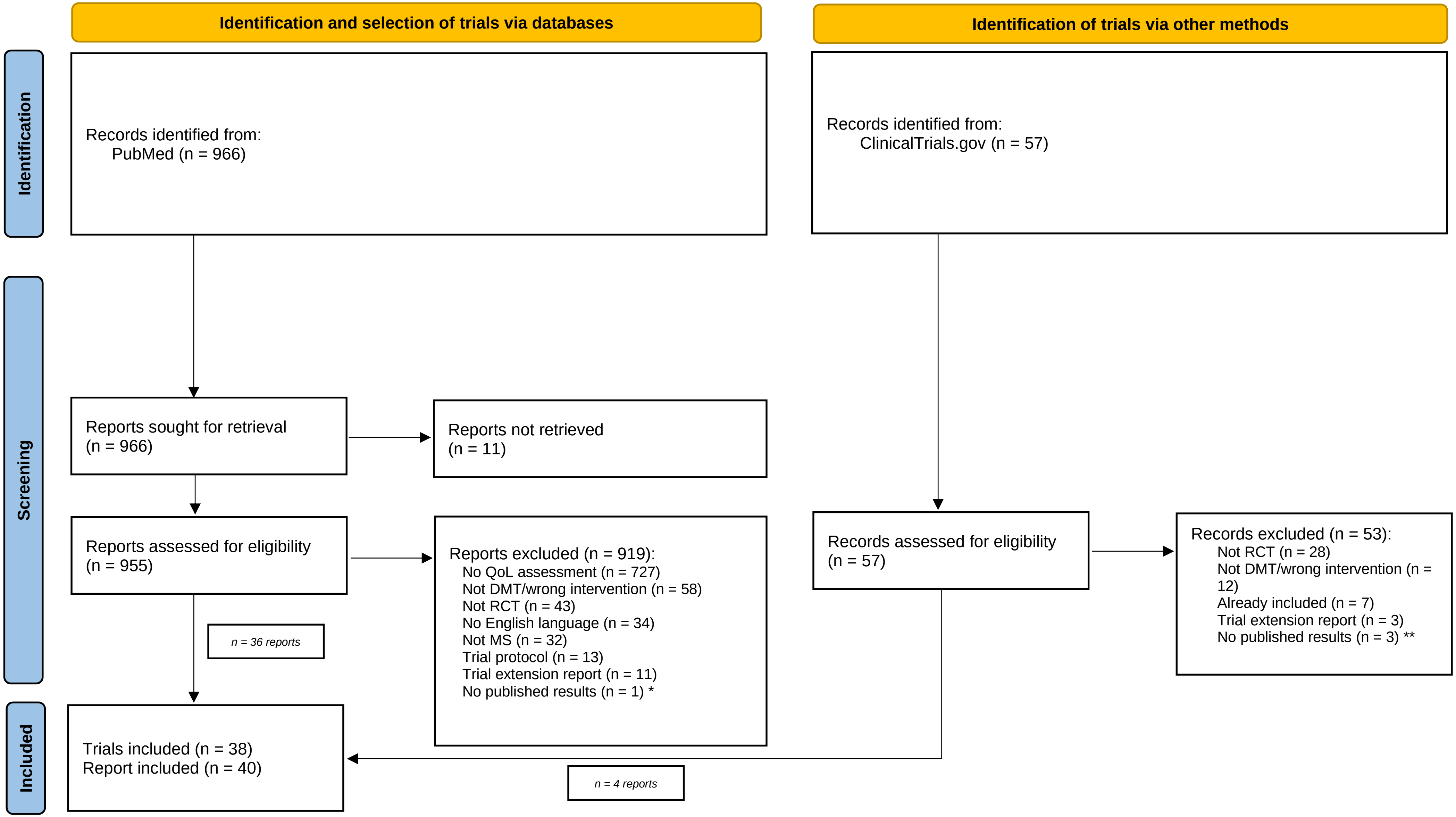

* For QOL measures, only subgroups were reported (i.e., participants who continued on their randomized treatment and those who restarted natalizumab earlier due to disease recurrence).

** Two trials being terminated with no results posted (other than specified in the registry): NCT00780455 and NCT01252355; one trial had prespecified QoL measures in the registry (NCT00559702), but did not refer to them in the identified publication.

Abbreviations: DMT=Disease modifying therapies; MS=Multiple Sclerosis; QoL=Quality of life; RCT=Randomized controlled trial.

Appendix 3. Raw trial data

The raw data that was extracted and analyzed for this study can be accessed via a separate spreadsheet containing separate sheets: trial data (n=38), measurement data (n=110), and QoL results (n=203).

Appendix 4.1. Re-calculated effect sizes (Hedges' g; n=89)

Trial	QoL scale	Experimental	Control	N	Mean change from baseline [95%CI]
CONFIDENCE	MSQLI mental	Lower frequency of glatiramer acetate administration	Higher frequency of glatiramer acetate administration	861	0.04 [−0.10; 0.17]
EPOCa	SF−36 physical	Fingolimod	DMT	54	−0.18 [−0.90; 0.53]
EPOCa	SF−36 mental	Fingolimod	DMT	54	−0.07 [−0.79; 0.64]
EPOCb	SF−36 physical	Fingolimod	DMT	291	0.14 [−0.14; 0.42]
EPOCb	SF−36 mental	Fingolimod	DMT	291	0.15 [−0.13; 0.43]
FREEDOMS II	EQ−5D health utility	Higher dose Fingolimod	Placebo	625	−0.05 [−0.21; 0.11]
FREEDOMS II	EQ−5D health utility	Lower dose Fingolimod	Placebo	527	−0.06 [−0.23; 0.12]
FREEDOMS II	EQ−5D health utility	Lower dose Fingolimod	Higher dose Fingolimod	642	−0.01 [−0.17; 0.15]
FREEDOMS II	EQ−5D VAS	Higher dose Fingolimod	Placebo	625	0.13 [−0.03; 0.29]
FREEDOMS II	EQ−5D VAS	Lower dose Fingolimod	Placebo	527	0.05 [−0.12; 0.22]
FREEDOMS II	EQ−5D VAS	Lower dose Fingolimod	Higher dose Fingolimod	642	−0.08 [−0.23; 0.08]
IMPACT	SF−36 mental	Interferon beta−1a	Placebo	257	0.21 [−0.03; 0.46]
IMPACT	SF−36 physical	Interferon beta−1a	Placebo	257	0.10 [−0.15; 0.34]
Nortvedt 1999 / Myhr 1999	SF−36 general health	Higher frequency of Peginterferon	Placebo	60	0.06 [−0.44; 0.56]
Nortvedt 1999 / Myhr 1999	SF−36 general health	Lower frequency of Peginterferon	Placebo	61	−0.02 [−0.52; 0.47]
Nortvedt 1999 / Myhr 1999	SF−36 mental	Higher frequency of Peginterferon	Placebo	60	−0.10 [−0.60; 0.40]
Nortvedt 1999 / Myhr 1999	SF−36 mental	Lower frequency of Peginterferon	Placebo	61	−0.14 [−0.64; 0.35]
Nortvedt 1999 / Myhr 1999	SF−36 pain	Higher frequency of Peginterferon	Placebo	60	−0.09 [−0.59; 0.42]
Nortvedt 1999 / Myhr 1999	SF−36 pain	Lower frequency of Peginterferon	Placebo	61	−0.12 [−0.61; 0.38]
Nortvedt 1999 / Myhr 1999	SF−36 physical	Higher frequency of Peginterferon	Placebo	60	−0.24 [−0.75; 0.26]
Nortvedt 1999 / Myhr 1999	SF−36 physical	Lower frequency of Peginterferon	Placebo	61	−0.22 [−0.72; 0.27]
Nortvedt 1999 / Myhr 1999	SF−36 role emotional	Higher frequency of Peginterferon	Placebo	60	0.01 [−0.49; 0.51]
Nortvedt 1999 / Myhr 1999	SF−36 role emotional	Lower frequency of Peginterferon	Placebo	61	0.03 [−0.47; 0.52]
Nortvedt 1999 / Myhr 1999	SF−36 role physical	Higher frequency of Peginterferon	Placebo	60	0.01 [−0.49; 0.51]
Nortvedt 1999 / Myhr 1999	SF−36 role physical	Lower frequency of Peginterferon	Placebo	61	−0.05 [−0.54; 0.45]
Nortvedt 1999 / Myhr 1999	SF−36 social	Higher frequency of Peginterferon	Placebo	60	−0.00 [−0.50; 0.50]
Nortvedt 1999 / Myhr 1999	SF−36 social	Lower frequency of Peginterferon	Placebo	61	−0.18 [−0.68; 0.31]
Nortvedt 1999 / Myhr 1999	SF−36 vitality	Higher frequency of Peginterferon	Placebo	60	0.01 [−0.50; 0.51]
Nortvedt 1999 / Myhr 1999	SF−36 vitality	Lower frequency of Peginterferon	Placebo	61	−0.08 [−0.58; 0.42]
NOVA	EQ−5D VAS	Natalizumab 6−week dosing	Natalizumab 4−week dosing	489	0.00 [−0.18; 0.18]
OPERA I	SF−36 physical	Ocrelizumab	Interferon Beta−1a	821	0.07 [−0.06; 0.21]
OPERA II	SF−36 physical	Ocrelizumab	Interferon Beta−1a	835	0.12 [−0.01; 0.26]
ORATORIO	SF−36 physical	Ocrelizumab	Placebo	732	0.04 [−0.12; 0.19]
RebiQoL	SF−36 physical	Rebif New Formulation Titrated	Rebif New Formulation Non Titrated	232	0.04 [−0.22; 0.30]
RebiQoL	SF−36 mental	Rebif New Formulation Titrated	Rebif New Formulation Non Titrated	232	−0.13 [−0.39; 0.12]
CARE−MS I	FAMS none	Alemtuzumab	Interferon beta−1a	533	0.09 [−0.10; 0.27]
CARE−MS II	FAMS none	Alemtuzumab	Interferon beta−1a	583	0.11 [−0.07; 0.28]
Montalban 2011	HAQUAMS none	high dose Fingolimod	Placebo	167	0.28 [−0.02; 0.59]
Montalban 2011	HAQUAMS none	high dose Fingolimod	low dose Fingolimod	176	−0.02 [−0.32; 0.27]
ADVANCE	MSIS−29 mental	Higher frequency of Peginterferon beta−1a	Placebo	1012	−0.01 [−0.13; 0.12]
ADVANCE	MSIS−29 physical	Higher frequency of Peginterferon beta−1a	Placebo	1012	0.08 [−0.04; 0.21]
ASCEND	MSIS−29 physical	Natalizumab	Placebo	858	0.13 [−0.00; 0.27]
NOVA	MSIS−29 mental	Natalizumab 6−week dosing	Natalizumab 4−week dosing	489	−0.03 [−0.20; 0.15]
NOVA	MSIS−29 physical	Natalizumab 6−week dosing	Natalizumab 4−week dosing	489	−0.04 [−0.21; 0.14]
ADVANCE	MSIS−29 mental	Lower frequency of Peginterferon beta−1a	Placebo	1000	−0.03 [−0.15; 0.10]
ADVANCE	MSIS−29 physical	Lower frequency of Peginterferon beta−1a	Placebo	1000	0.01 [−0.12; 0.13]
ADVANCE	MSIS−29 mental	Lower frequency of Peginterferon beta−1a	Higher frequency of Peginterferon beta−1a	1012	−0.02 [−0.14; 0.10]
ADVANCE	MSIS−29 physical	Lower frequency of Peginterferon beta−1a	Higher frequency of Peginterferon beta−1a	1012	−0.08 [−0.20; 0.05]
RADIANCE	MSQOL−54 mental	high dose Ozanimod	Interferon beta−1a	874	0.02 [−0.11; 0.15]
RADIANCE	MSQOL−54 mental	low dose Ozanimod	Interferon beta−1a	880	0.04 [−0.09; 0.17]
RADIANCE	MSQOL−54 mental	high dose Ozanimod	low dose Ozanimod	872	−0.02 [−0.16; 0.11]
RADIANCE	MSQOL−54 physical	high dose Ozanimod	low dose Ozanimod	872	−0.03 [−0.17; 0.10]
CONFIDENCE	MSQLI fatigue	Lower frequency of Glatiramer Acetate	Higher frequency of Glatiramer Acetate	847	0.02 [−0.11; 0.16]
IMPACT	MSQLI bladder control	Interferon beta−1a	Placebo	259	0.14 [−0.10; 0.39]
IMPACT	MSQLI bowel control	Interferon beta−1a	Placebo	260	0.10 [−0.15; 0.34]
IMPACT	MSQLI pain	Interferon beta−1a	Placebo	262	0.21 [−0.03; 0.46]
IMPACT	MSQLI sexual satisfaction	Interferon beta−1a	Placebo	190	0.27 [−0.01; 0.56]
IMPACT	MSQLI visual impairment	Interferon beta−1a	Placebo	261	0.04 [−0.20; 0.29]
RebiQoL	MSTCQ none	Rebif New Formulation Non Titrated	Rebif New Formulation Non Titrated	230	−0.17 [−0.43; 0.09]
RELIEF	MusiQoL none	Evening administration	Morning administration	153	−0.26 [−0.57; 0.06]
NOVA	Neuro−QoL fatigue	Natalizumab 6−week dosing	Natalizumab 4−week dosing	489	−0.05 [−0.22; 0.13]
EPOCc	SF−36 mental	Fingolimod	Injectable DMT	1052	0.21 [0.07; 0.35]
EPOCc	SF−36 physical	Fingolimod	Injectable DMT	1052	0.19 [0.05; 0.33]
IMPACT	MSQLI mental	Interferon beta−1a	Placebo	265	0.29 [0.05; 0.53]
IMPACT	MSQLI perceived deficits	Interferon beta−1a	Placebo	264	0.25 [0.01; 0.50]
IMPACT	MSQLI social support	Interferon beta−1a	Placebo	258	0.27 [0.03; 0.52]
TOWER	SF−36 mental	Higher dose of Teriflunomide	Lower dose of Teriflunomide	777	1.02 [0.87; 1.17]
TOWER	SF−36 physical	Higher dose of Teriflunomide	Lower dose of Teriflunomide	777	0.71 [0.56; 0.85]
TOWER	SF−36 mental	Lower dose of Teriflunomide	Placebo	795	1.52 [1.36; 1.67]
TOWER	SF−36 mental	Higher dose of Teriflunomide	Placebo	758	2.46 [2.28; 2.65]
TOWER	SF−36 physical	Lower dose of Teriflunomide	Placebo	795	1.68 [1.52; 1.84]
TOWER	SF−36 physical	Higher dose of Teriflunomide	Placebo	758	2.34 [2.15; 2.52]
Montalban 2011	HAQUAMS none	low dose Fingolimod	Placebo	177	0.35 [0.05; 0.65]
GLACIER	MSIS−29 mental	Glatiramer acetate 40mg	Glatiramer acetate 20mg	177	2.41 [2.02; 2.80]
GLACIER	MSIS−29 physical	Glatiramer acetate 40mg	Glatiramer acetate 20mg	177	1.38 [1.05; 1.71]
GOLDEN	MSQOL−54 mental	Fingolimod	Interferon beta 1b	108	2.89 [2.31; 3.48]
GOLDEN	MSQOL−54 physical	Fingolimod	Interferon beta 1b	108	1.68 [1.20; 2.17]
RADIANCE	MSQOL−54 physical	low dose Ozanimod	Interferon beta−1a	880	0.17 [0.04; 0.31]
RADIANCE	MSQOL−54 physical	high dose Ozanimod	Interferon beta−1a	874	0.14 [0.01; 0.27]
IMPACT	MSQLI fatigue	Interferon beta−1a	Placebo	267	0.30 [0.06; 0.54]
Panitch 2004	MSQLI none	Higher dose of Interferon−beta 1b	Lower dose of Interferon−beta 1b	619	1.36 [1.19; 1.54]
Panitch 2004	MSQLI none	Higher dose of Interferon−beta 1b	Placebo	614	0.80 [0.64; 0.97]
Panitch 2004	MSQLI none	Lower dose of Interferon−beta 1b	Placebo	613	−0.50 [−0.66; −0.34]
SUNBEAM	MSQOL mental	Lower dose of Ozanimod	Interferon beta−1a	899	1.52 [1.37; 1.67]
SUNBEAM	MSQOL mental	Higher dose of Ozanimod	Interferon beta−1a	894	2.48 [2.30; 2.65]
SUNBEAM	MSQOL mental	Higher dose of Ozanimod	Lower dose of Ozanimod	897	1.01 [0.87; 1.15]
SUNBEAM	MSQOL physical	Lower dose of Ozanimod	Interferon beta−1a	893	1.71 [1.56; 1.86]
SUNBEAM	MSQOL physical	Higher dose of Ozanimod	Interferon beta−1a	888	2.37 [2.20; 2.54]
SUNBEAM	MSQOL physical	Higher dose of Ozanimod	Lower dose of Ozanimod	891	0.72 [0.58; 0.85]

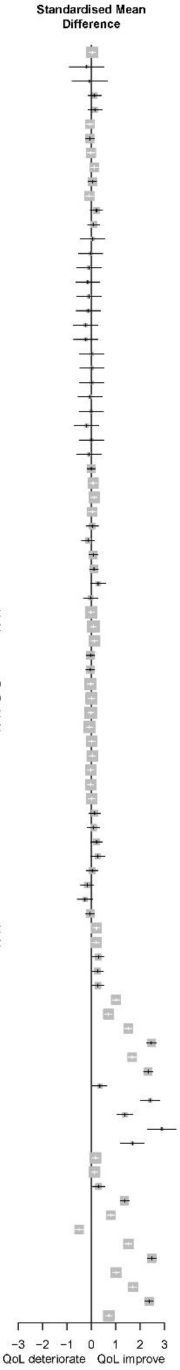

Abbreviations: CI=Confidence interval; N=Number of randomized participants; QoL=Quality of life.

Appendix 4.2. Re-calculated effect sizes (Cohen's d; n=89)

Trial	QoL scale	Experimental	Control	N	Mean change from baseline [95%CI]
CONFIDENCE	MSQLI mental	Lower frequency of glatiramer acetate administration	Higher frequency of glatiramer acetate administration	861	0.04 [−0.10; 0.17]
EPOCa	SF−36 physical	Fingolimod	DMT	54	−0.19 [−0.90; 0.53]
EPOCa	SF−36 mental	Fingolimod	DMT	54	−0.07 [−0.79; 0.64]
EPOCb	SF−36 physical	Fingolimod	DMT	291	0.14 [−0.14; 0.42]
EPOCb	SF−36 mental	Fingolimod	DMT	291	0.15 [−0.13; 0.43]
FREEDOMS II	EQ−5D health utility	Higher dose Fingolimod	Placebo	625	−0.05 [−0.21; 0.11]
FREEDOMS II	EQ−5D health utility	Lower dose Fingolimod	Placebo	527	−0.06 [−0.23; 0.11]
FREEDOMS II	EQ−5D health utility	Lower dose Fingolimod	Higher dose Fingolimod	642	−0.01 [−0.17; 0.15]
FREEDOMS II	EQ−5D VAS	Higher dose Fingolimod	Placebo	625	0.13 [−0.03; 0.29]
FREEDOMS II	EQ−5D VAS	Lower dose Fingolimod	Placebo	527	0.05 [−0.12; 0.22]
FREEDOMS II	EQ−5D VAS	Lower dose Fingolimod	Higher dose Fingolimod	642	−0.08 [−0.23; 0.08]
IMPACT	SF−36 mental	Interferon beta−1a	Placebo	257	0.21 [−0.03; 0.46]
IMPACT	SF−36 physical	Interferon beta−1a	Placebo	257	0.10 [−0.15; 0.34]
Nortvedt 1999 / Myhr 1999	SF−36 general health	Higher frequency of Peginterferon	Placebo	60	0.06 [−0.45; 0.57]
Nortvedt 1999 / Myhr 1999	SF−36 general health	Lower frequency of Peginterferon	Placebo	61	−0.02 [−0.53; 0.48]
Nortvedt 1999 / Myhr 1999	SF−36 mental	Higher frequency of Peginterferon	Placebo	60	−0.10 [−0.61; 0.41]
Nortvedt 1999 / Myhr 1999	SF−36 mental	Lower frequency of Peginterferon	Placebo	61	−0.14 [−0.65; 0.36]
Nortvedt 1999 / Myhr 1999	SF−36 pain	Higher frequency of Peginterferon	Placebo	60	−0.09 [−0.59; 0.42]
Nortvedt 1999 / Myhr 1999	SF−36 pain	Lower frequency of Peginterferon	Placebo	61	−0.12 [−0.62; 0.38]
Nortvedt 1999 / Myhr 1999	SF−36 physical	Higher frequency of Peginterferon	Placebo	60	−0.25 [−0.76; 0.26]
Nortvedt 1999 / Myhr 1999	SF−36 physical	Lower frequency of Peginterferon	Placebo	61	−0.23 [−0.73; 0.28]
Nortvedt 1999 / Myhr 1999	SF−36 role emotional	Higher frequency of Peginterferon	Placebo	60	0.01 [−0.49; 0.52]
Nortvedt 1999 / Myhr 1999	SF−36 role emotional	Lower frequency of Peginterferon	Placebo	61	0.03 [−0.47; 0.53]
Nortvedt 1999 / Myhr 1999	SF−36 role physical	Higher frequency of Peginterferon	Placebo	60	0.01 [−0.50; 0.52]
Nortvedt 1999 / Myhr 1999	SF−36 role physical	Lower frequency of Peginterferon	Placebo	61	−0.05 [−0.55; 0.46]
Nortvedt 1999 / Myhr 1999	SF−36 social	Higher frequency of Peginterferon	Placebo	60	−0.00 [−0.51; 0.50]
Nortvedt 1999 / Myhr 1999	SF−36 social	Lower frequency of Peginterferon	Placebo	61	−0.19 [−0.69; 0.32]
Nortvedt 1999 / Myhr 1999	SF−36 vitality	Higher frequency of Peginterferon	Placebo	60	0.01 [−0.50; 0.51]
Nortvedt 1999 / Myhr 1999	SF−36 vitality	Lower frequency of Peginterferon	Placebo	61	−0.08 [−0.58; 0.42]
NOVA	EQ−5D VAS	Natalizumab 6−week dosing	Natalizumab 4−week dosing	489	0.00 [−0.18; 0.18]
OPERA I	SF−36 physical	Ocrelizumab	Interferon Beta−1a	821	0.07 [−0.06; 0.21]
OPERA II	SF−36 physical	Ocrelizumab	Interferon Beta−1a	835	0.12 [−0.01; 0.26]
ORATORIO	SF−36 physical	Ocrelizumab	Placebo	732	0.04 [−0.12; 0.19]
RebiQoL	SF−36 physical	Rebif New Formulation Titrated	Rebif New Formulation Non Titrated	232	0.04 [−0.22; 0.30]
RebiQoL	SF−36 mental	Rebif New Formulation Titrated	Rebif New Formulation Non Titrated	232	−0.13 [−0.39; 0.12]
CARE−MS I	FAMS none	Alemtuzumab	Interferon beta−1a	533	0.09 [−0.10; 0.27]
CARE−MS II	FAMS none	Alemtuzumab	Interferon beta−1a	583	0.11 [−0.07; 0.29]
Montalban 2011	HAQUAMS none	high dose Fingolimod	Placebo	167	0.29 [−0.02; 0.59]
Montalban 2011	HAQUAMS none	high dose Fingolimod	low dose Fingolimod	176	−0.02 [−0.32; 0.27]
ADVANCE	MSIS−29 mental	Higher frequency of Peginterferon beta−1a	Placebo	1012	−0.01 [−0.13; 0.12]
ADVANCE	MSIS−29 physical	Higher frequency of Peginterferon beta−1a	Placebo	1012	0.08 [−0.04; 0.21]
ASCEND	MSIS−29 physical	Natalizumab	Placebo	858	0.13 [−0.00; 0.27]
NOVA	MSIS−29 mental	Natalizumab 6−week dosing	Natalizumab 4−week dosing	489	−0.03 [−0.20; 0.15]
NOVA	MSIS−29 physical	Natalizumab 6−week dosing	Natalizumab 4−week dosing	489	−0.04 [−0.21; 0.14]
ADVANCE	MSIS−29 mental	Lower frequency of Peginterferon beta−1a	Placebo	1000	−0.03 [−0.15; 0.10]
ADVANCE	MSIS−29 physical	Lower frequency of Peginterferon beta−1a	Placebo	1000	0.01 [−0.12; 0.13]
ADVANCE	MSIS−29 mental	Lower frequency of Peginterferon beta−1a	Higher frequency of Peginterferon beta−1a	1012	−0.02 [−0.14; 0.10]
ADVANCE	MSIS−29 physical	Lower frequency of Peginterferon beta−1a	Higher frequency of Peginterferon beta−1a	1012	−0.08 [−0.20; 0.05]
RADIANCE	MSQOL−54 mental	high dose Ozanimod	Interferon beta−1a	874	0.02 [−0.11; 0.15]
RADIANCE	MSQOL−54 mental	low dose Ozanimod	Interferon beta−1a	880	0.04 [−0.09; 0.17]
RADIANCE	MSQOL−54 mental	high dose Ozanimod	low dose Ozanimod	872	−0.02 [−0.16; 0.11]
RADIANCE	MSQOL−54 physical	high dose Ozanimod	low dose Ozanimod	872	−0.03 [−0.17; 0.10]
CONFIDENCE	MSQLI fatigue	Lower frequency of Glatiramer Acetate	Higher frequency of Glatiramer Acetate	847	0.02 [−0.11; 0.16]
IMPACT	MSQLI bladder control	Interferon beta−1a	Placebo	259	0.14 [−0.10; 0.39]
IMPACT	MSQLI bowel control	Interferon beta−1a	Placebo	260	0.10 [−0.15; 0.34]
IMPACT	MSQLI pain	Interferon beta−1a	Placebo	262	0.21 [−0.03; 0.46]
IMPACT	MSQLI sexual satisfaction	Interferon beta−1a	Placebo	190	0.27 [−0.01; 0.56]
IMPACT	MSQLI visual impairment	Interferon beta−1a	Placebo	261	0.04 [−0.20; 0.29]
RebiQoL	MSTCQ none	Rebif New Formulation Non Titrated	Rebif New Formulation Non Titrated	230	−0.17 [−0.43; 0.09]
RELIEF	MusiQoL none	Evening administration	Morning administration	153	−0.26 [−0.57; 0.06]
NOVA	Neuro−QoL fatigue	Natalizumab 6−week dosing	Natalizumab 4−week dosing	489	−0.05 [−0.22; 0.13]
EPOCc	SF−36 mental	Fingolimod	Injectable DMT	1052	0.21 [0.07; 0.35]
EPOCc	SF−36 physical	Fingolimod	Injectable DMT	1052	0.19 [0.05; 0.33]
IMPACT	MSQLI mental	Interferon beta−1a	Placebo	265	0.29 [0.05; 0.53]
IMPACT	MSQLI perceived deficits	Interferon beta−1a	Placebo	264	0.26 [0.01; 0.50]
IMPACT	MSQLI social support	Interferon beta−1a	Placebo	258	0.27 [0.03; 0.52]
TOWER	SF−36 mental	Higher dose of Teriflunomide	Lower dose of Teriflunomide	777	1.02 [0.87; 1.17]
TOWER	SF−36 physical	Higher dose of Teriflunomide	Lower dose of Teriflunomide	777	0.71 [0.56; 0.85]
TOWER	SF−36 mental	Lower dose of Teriflunomide	Placebo	795	1.52 [1.36; 1.68]
TOWER	SF−36 mental	Higher dose of Teriflunomide	Placebo	758	2.47 [2.28; 2.66]
TOWER	SF−36 physical	Lower dose of Teriflunomide	Placebo	795	1.68 [1.52; 1.84]
TOWER	SF−36 physical	Higher dose of Teriflunomide	Placebo	758	2.34 [2.15; 2.52]
Montalban 2011	HAQUAMS none	low dose Fingolimod	Placebo	177	0.35 [0.05; 0.65]
GLACIER	MSIS−29 mental	Glatiramer acetate 40mg	Glatiramer acetate 20mg	177	2.42 [2.03; 2.81]
GLACIER	MSIS−29 physical	Glatiramer acetate 40mg	Glatiramer acetate 20mg	177	1.38 [1.05; 1.71]
GOLDEN	MSQOL−54 mental	Fingolimod	Interferon beta 1b	108	2.91 [2.33; 3.50]
GOLDEN	MSQOL−54 physical	Fingolimod	Interferon beta 1b	108	1.70 [1.21; 2.18]
RADIANCE	MSQOL−54 physical	low dose Ozanimod	Interferon beta−1a	880	0.17 [0.04; 0.31]
RADIANCE	MSQOL−54 physical	high dose Ozanimod	Interferon beta−1a	874	0.14 [0.01; 0.27]
IMPACT	MSQLI fatigue	Interferon beta−1a	Placebo	267	0.30 [0.06; 0.55]
Panitch 2004	MSQLI none	Higher dose of Interferon−beta 1b	Lower dose of Interferon−beta 1b	619	1.37 [1.19; 1.54]
Panitch 2004	MSQLI none	Higher dose of Interferon−beta 1b	Placebo	614	0.81 [0.64; 0.97]
Panitch 2004	MSQLI none	Lower dose of Interferon−beta 1b	Placebo	613	−0.50 [−0.66; −0.34]
SUNBEAM	MSQOL mental	Lower dose of Ozanimod	Interferon beta−1a	899	1.52 [1.38; 1.67]
SUNBEAM	MSQOL mental	Higher dose of Ozanimod	Interferon beta−1a	894	2.48 [2.31; 2.66]
SUNBEAM	MSQOL mental	Higher dose of Ozanimod	Lower dose of Ozanimod	897	1.01 [0.88; 1.15]
SUNBEAM	MSQOL physical	Lower dose of Ozanimod	Interferon beta−1a	893	1.71 [1.56; 1.86]
SUNBEAM	MSQOL physical	Higher dose of Ozanimod	Interferon beta−1a	888	2.37 [2.20; 2.55]
SUNBEAM	MSQOL physical	Higher dose of Ozanimod	Lower dose of Ozanimod	891	0.72 [0.58; 0.85]

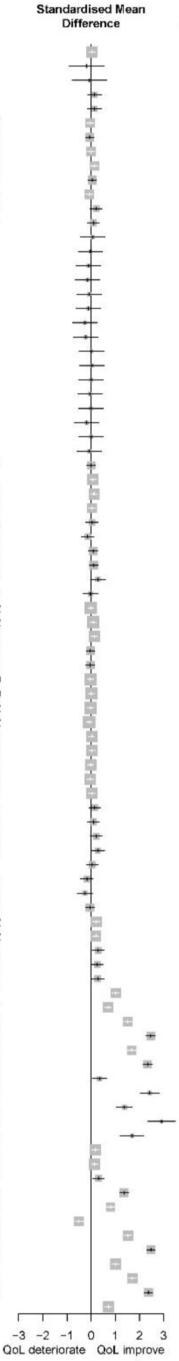

Abbreviations: CI=Confidence interval; N=Number of randomized participants; QoL=Quality of life.
